# A Simple Chi-Square Statistic for Testing Homogeneity of Zero-Inflated Distributions

**DOI:** 10.4236/ojs.2015.56050

**Published:** 2015-10-13

**Authors:** William D. Johnson, Jeffrey H. Burton, Robbie A. Beyl, Jacob E. Romer

**Affiliations:** Department of Biostatistics, Pennington Biomedical Research Center, Louisiana State University, Baton Rouge, LA, USA

**Keywords:** Asymptotic Chi-Square Test, Equality of Quantiles, Large Sample Test, Nonparametric Test, Percentile Profiles, Zero-Inflated Distributions

## Abstract

Zero-inflated distributions are common in statistical problems where there is interest in testing homogeneity of two or more independent groups. Often, the underlying distribution that has an inflated number of zero-valued observations is asymmetric, and its functional form may not be known or easily characterized. In this case, comparisons of the groups in terms of their respective percentiles may be appropriate as these estimates are nonparametric and more robust to outliers and other irregularities. The median test is often used to compare distributions with similar but asymmetric shapes but may be uninformative when there are excess zeros or dissimilar shapes. For zero-inflated distributions, it is useful to compare the distributions with respect to their proportion of zeros, coupled with the comparison of percentile profiles for the observed non-zero values. A simple chi-square test for simultaneous testing of these two components is proposed, applicable to both continuous and discrete data. Results of simulation studies are reported to summarize empirical power under several scenarios. We give recommendations for the minimum sample size which is necessary to achieve suitable test performance in specific examples.

## 1. Introduction

Zero-inflated distributions—a mixture of a point distribution and some other non-zero distribution (s)—are common in biomedical studies. Typically, there is interest in making comparisons between such groups, as in testing the homogeneity of distributions across treatment groups or other populations of interest. In addition to excess zeros, analysis is made more difficult by the distribution of the non-zero data, which is most likely asymmetric or multimodal. Zhang *et al*. [1] studied the properties of six tests for equality of zero-inflated continuous distributions, finding some of them inaccurate when the parametric distribution was not specified correctly. Typically, zero-valued observations are relevant in a way that results from some important characteristics or behavior. Thus, the ideal statistical test for comparing zero-inflated distributions would simultaneously test for equality of both 1) the proportion of zero values and 2) the non-zero distribution, and do so with little to no assumptions regarding the data. The proposed test, an extension/special application of the percentile test developed by Johnson *et al.* [2], can be used to test the equality of both components of zero-inflated distributions with respect to the distributions’ percentiles (quantiles). We use the term *percentile profile* to denote a set of percentiles that we use collectively as a metric to compare groups.

Zero-inflated distributions arise under a variety of circumstances. For example, some laboratory procedures may be unable to detect values below a certain threshold and, by default, are recorded as zero, the lowest detection level, or some other value. Another scenario may be that subjects from a portion of the population do not exhibit symptoms (their responses are recorded as zero since their response cannot be measured) while others do have symptoms and some measurements are taken. Data may be categorized and categories may be combined to simplify the analysis. For instance, subjects below a certain age may be coded with a single number which would censor the observed data at a point and create a point distribution at this value. Count data with excess zeros are one of the most prevalent examples of zero-inflated distributions with zero-inflated Poisson and negative binomial models used extensively in a variety of fields [3]–[5]. Despite the prevalence of zero-inflated distributions, there are very few explicit tests for homogeneity, especially nonparametric tests that are appropriate for either continuous or discrete data.

Lachenbruch [6] [7] proposed two-part models to test for equality of distributions with respect to probabilities of zeros and location parameters in continuous distributions for the non-zero data. A test statistic incorporating a binomial test and either the t-test, Kolmogorov-Smirnov test, or Wilcoxon test was created to test equality of two zero-inflated distributions. Because excess zeros are typical of count data, several tests for zero-inflated Poisson distributions have been developed using likelihood ratio methods [8] [9] and exact tests [10], but these assume the data follow a Poisson distribution, a tenet that may be problematic. If a parametric distribution is assumed as in [1], standard tests such as the likelihood ratio and Wald tests do not perform well if the precise functional form of the distribution is unknown or mis-specified due to inflation of Type I errors. To correct for this, Wu *et al.* [11] used a permutation test to adjust Type I errors in the likelihood ratio and Wald tests. Also, Hallstrom [12] used a truncated Wilcoxon test where the zero observations were removed.

As an extension of the procedure outlined in [2], the proposed test possesses good large-sample properties. Not only is the test especially useful when dealing with asymmetric data or in cases where the distribution cannot be assumed, it can be used with either continuous or discrete data. The test is also flexible because profiles of any size can be simultaneously compared for two or more populations. Unlike a single parameter test for equality of location, such as the median test, it is more informative to summarize the characteristics of the respective shapes across the entire range of the distributions. By recognizing that the proportion of zeros within a sample may be treated as another percentile, we incorporate this into the same procedure of testing equality of percentiles, thereby simultaneously testing the equality of the proportion of zeros as well as the specified percentile profile with respect to the non-zero distribution.

The proposed general strategy for comparing percentile profiles [2] can be used in a wide class of applications where the primary outcome has a zero-inflated distribution. Section 2 describes the procedure for invoking the general percentile test and details needed to compare zero-inflated distributions. Results of selected simulation studies performed to investigate empirical power are presented in Section 3 followed by two illustrative examples in Section 4.

## 2. Test Procedure

### 2.1. General Test for Homogeneity of Percentile Profiles

Let *Q_h_* = (*Q_h_*_1_, *Q_h_*_2_, ···, *Q_hp_*), denote a profile of percentiles in population *h*, where *h* = 1, ···, *K*. Suppose random samples are available from each of the *K* populations and we wish to test the null hypothesis that the profiles are identical for all *K* populations; that is, we wish to test *H*_0_: *Q*_1_ = *Q*_2_ = ··· = *Q_K_* against the alternative, *H*_1_: one or more inequalities exist such that *Q_hj_* ≠ *Q_ij_* for at least one *h* ≠ *i* where *h*,*i* = 1, 2, ···, *K* and at least one *j* = 1, 2, ···, *p*. Thus, the test is a simultaneous test of equality of the profiles across the *K* populations. The following procedure is the general percentile test described in [2]:

1. Combine the *K* samples and calculate the combined sample percentile estimate of *Q̄* = (*Q̄*_1_, *Q̄*_2_, ···, *Q̄_p_*). Denote the combined sample percentile profile estimate as *q̄* = (*q̄*_1_, *q̄*_2_, ···, *q̄_p_*).
2. For each of the *K* samples, sort the observations into categories or bins with cutoffs based on the combined sample percentile estimates, *q̄*, to create *p* + 1 bins of data. For example: bin_1_ = {all observations ≤ *q̄*_1_}, bin_2_ = { *q̄*_1_ < all observations ≤ *q̄*_2_}, ···, bin*_p_* = {*q̄_p_*_−1_ < all observations ≤ *q̄_p_*}, bin*_p_*_+1_ = {all observations > *q̄_p_*}.
3. Arrange the categorized data from Step (2) in a *K* × (*p* + 1) contingency table where each row, respectively, consists of *p* + 1 sorted sets of observations for one of the samples.
4. Perform the test of homogeneity of the percentile profiles in terms of Pearson’s chi-square statistic with *p*(*K* − 1) degrees of freedom.

To illustrate, suppose we are given three samples and we wish to test *H*_0_: *Q*_1_ = *Q*_2_ = *Q*_3_ where the percentile profile *Q_h_* = (25, 50, 75), *h* = 1, 2, 3. Further, suppose estimates of these percentiles were found to be *q*_1_ = (4.6, 6.4, 10.4), *q*_2_ = (6.2, 8.3, 12.3), and *q*_3_ = (4.8, 7.9, 10.6), respectively, in samples from the three distributions. The combined sample estimates of these percentiles are *q̄*_1_ =5.1, *q̄*_2_ =7.7 and *q̄*_3_ =11.1, respectively, with the resulting 3 × 4 contingency table from step 2 as shown in Table 1. For the data in Table 1, the chi-square value is about 14.1 with six degrees of freedom (p-value = 0.029). Therefore we would reject *H*_0_ and conclude that the percentile profiles are not homogeneous across the three distributions.

### 2.2. Comparing Zero-Inflated Distributions

Let *D* be a zero-inflated distribution; *D* = *πg* + (1 − *π*)*f*, where *π* is the probability of an observation being in the point distribution of zeros, *g*, and *f* is the non-zero distribution (*g* is not necessarily located at 0, but could be any value at the minimum or maximum in the domain). Suppose we are given zero-inflated sample data from *K* independent populations and we wish to test the null hypothesis that the underlying distributions are all identical. Let *D_i_* = *π_i_g* + (1 − *π_i_*)*f_i_*; *i* =1, ···, *K*, and consider *H*_0_: *D*_1_ = *D*_2_ = ··· = *D_K_*. It is assumed that *g* is equal in all populations because it is a parameter of a specific process that produces *D*, such as limits on a measurement device. In order for the null hypothesis (all *D_i_* are identical) to be false, at least one of *π_i_* or *f_i_* must be unequal for at least one pair of distributions. Note that *D* could also contain two point distributions, one at the minimum value and one at the maximum in the domain. In this case, *D_i_* = *π_i_*_1_*g_L_* +*π_i_*_2_*g_U_* + (1 − *π_i_*_1_ − *π_i_*_2_)*f_i_*, where *g_L_* is the point distribution at the minimum value and *g_U_* is the point distribution at the maximum value, with probability *π_i_*_1_ and *π_i_*_2_, respectively. The focus of this paper is on point distributions at the minimum of the domain although the procedure for point distributions at the maximum is identical.

Consider, for example, testing the equality of groups, such as race/gender cohorts, with respect to their degree of tobacco smoke exposure assessed in terms of the tobacco biomarker, cotinine (details later). Within the groups, there may be some people with undetectable or nonexistent levels of cotinine; we will consider these non-smokers who have not been exposed to measurable amounts of secondary smoke. Mixed with this unexposed population (within the groups) are those who either currently smoke, or have a history of smoking or being exposed to measurable amounts of second-hand smoke and thus have cotinine levels above the detection limit. We will consider these people to be exposed to smoking either directly or through people around them smoking. It is informative to test the equality of groups comprised of mixtures of exposed and unexposed populations-the proportion unexposed and the percentile profiles that reflect the severity of exposure in each group.

The general percentile test outlined in the previous section can be used to simultaneously test the equality of proportions of zeros (non-smokers) in addition to the equality of the percentile profiles (distributions of smokers) across populations. Let *X_i_* denote the observed cotinine assessment for a person who is randomly selected from the *i*^th^ population, and let *π_i_* (*i* = 1, ···, *K*) be the proportion of zeros in the *i*^th^ population. *P*[*X_i_* ≤ 0] = *π_i_* and the value of any percentile less than 100 × *π_i_* is 0.

Thus, 0 could be considered another percentile estimate-the sample estimate of the 100 × *π_i_*^th^ percentile. Since any percentile less than 100 × *π_i_* is equal to 0, we can select an arbitrary percentile such that each population’s estimate is 0. However, because we use the combined sample percentile estimate to create bins for the contingency table, we must select a percentile less than the combined sample proportion of zeros, denoted *π̄*. Essentially, the purpose is to create a bin in the contingency table (from step 3, Section 2.1) where all values equal to 0 are placed.

Suppose we wish to test the equality of a percentile profile *Q* across *K* populations with the proportion of zeros in the combined population equal to *π̄*. Adding an arbitrary small number *Q_z_* ≤ 100×*π̄* to *Q* and proceeding through the steps from Section 2.1 will test the equality of the proportion of zeros as well as the original percentile profile of interest. Denote the combined sample percentile estimates as (*g*, *q̄*_1_, ···, *q̄_p_*) where *g* is the value of the point distribution (0) and (*q̄*_1_, ···, *q̄_p_*) are the estimates of *Q* for the combined samples. For each sample, all observations equal to 0 will be placed into the first bin, all observations in the interval (0, *q̄*_1_] will be placed into the second bin, and so on. For zero-inflated distributions, the percentiles of interest in *Q* must be selected with care. If *Q* ≤ 100×*π̄* is chosen, there will be multiple percentile estimates in *q̄* equal to 0 and redundant percentile estimates will lead to empty bins (all observations ≤ 0 will be placed into the first bin by default). To avoid this, only percentiles greater than the combined proportion of zeros should be selected. Furthermore, there should be adequate space between *π̄* and *Q*_1_, the first non-zero percentile, to ensure sufficient expected frequencies (under the null hypothesis) used to calculate the chi-square test (typically at least five in each cell of the contingency table).

For example, suppose the data used to create Table 1 had a large number of zeros mixed with some non-zero distribution where the proportion of zeros in populations 1 – 3 (denoted *π*_1_, *π*_2_, and *π*_3_) are used to provide sample estimates equal to 100 × 15/220 = 6.8, 100 × 28/196 = 14.3, and 100 × 42/208 = 20.2, respectively. The combined sample estimate of the proportion of zeros *π̄* in the combined population is equal to 100 × 85/624, or 13.6. We wish to test *H*_0_: *Q*_1_ = *Q*_2_ = *Q*_3_ where *Q* = (25, 50, 75) and simultaneously test the equality of *π*_1_, *π*_2_, and *π*_3_. We simply add another bin to the contingency table for observations equal to zero—“Bin 1” in Table 2 (combining bins one and two in Table 2 would be equivalent to Table 1). To do this, we add a percentile *Q_z_* ≤ 100×*π̄* to *Q*, so that we now use *Q* = (1, 25, 50, 75) to proceed through the steps. For the data in Table 2, the chi-square statistic is about 64.1 with eight degrees of freedom (p-value < 0.0001). We would reject *H*_0_ and conclude that the percentile profiles of the 1^st^, 25^th^, 50^th^ and 75^th^ percentiles are not homogeneous between the three distributions. Thus it is a simultaneous test of the equality of the proportion of zeros as well as the percentile profile.

As previously mentioned the point distribution of interest is not restricted to 0 but can be any value that is the minimum of the data. Also, this procedure is applicable to point distributions at the maximum value. Instead of adding a percentile near 0 to *Q*, an arbitrary percentile close to 100 can be chosen, such as 99. If *π̄* is the combined population proportion of observations equal to *u*, the point distribution of the maximum value, we would add a *Q_z_* ≥ 100×*π̄* to *Q* while ensuring that *Q_p_* < 100×*π̄* (with adequate spacing). As in the case of having a minimum-value point distribution, we avoid redundant percentile estimates of the combined samples. Suppose we were given data with point distributions at both the minimum and maximum value. In this case, we could test homogeneity of proportions of both point distributions as well as the percentile profile with *Q* = (1, *Q*_1_, ···, *Q_p_*, 99).

## 3. Power Simulations

Some asymptotic properties of the percentile test with zero inflated distributions were investigated for both continuous and discrete data. Power simulations were conducted for several scenarios by varying the proportion of zeros and/or the properties of the non-zero distributions. For this paper, point distributions at zero mixed with non-zero gamma distributions were considered for the continuous case (Table 3). Poisson distributions were used to illustrate the discrete case (Table 4). The subscripts for the parameters in the tables refer to the respective samples with sample “2” having constant values for the non-zero distribution. For the continuous case, sample “2” has shape parameter that is held at *α*_2_ = 2, and the scale parameter held at *β*_2_ = 2. For the discrete case, the parameter of sample “2” is held constant at *λ*_2_ = 5. Although parametric distributions were used to generate data, there are no assumptions or requirements for the distribution of the data. Gamma and Poisson distributions were used as convenient tools for generating skewed distributions for the non-zero component. The procedure works equally well for distributions with mixtures of several non-zero distributions.

As a multivariate problem, finding an exact probability of the test or expressing some measure of difference between distributions is a challenge. The power of the test is a complex function of the difference in probability of observing a zero, combined with the probabilities of the non-zero values being placed in the particular bins given the probability of a certain proportion of zeros. Furthermore, the choice of percentiles affects the power of the test as the relative proportions of bins affects the chi-square test. However, we attempt to relate the power of the test with certain characteristics of the data and choice of percentiles in situations that may be common in applications. All power estimates are based on 10,000 replicate samples and all procedures were programmed and carried out with R 3.1.2.

The results of the simulations using data generated from a mixture of a gamma distribution with zeros and testing the profile *Q* = (1, 50, 75, 90) are presented in Table 3. Although limited in scope, the results highlight some important features of the test. Empirical alpha (first column of power results in boldface) is adequate by sample size 50 and converges to 0.05 in sample sizes somewhere between 50 and 100. The power of the test is determined by interaction between the difference in the true ratio between rows in the contingency table (determined by the difference in the distributions and probability of zeros) and the relative proportion of each bin as well as sample size. In general, power is maximized when the relative proportion of bins corresponds with the difference in the row profiles. The size of the bin is determined by the percentile profile to be tested, *Q*, with the ratio of observations from each row within the bin determined by the characteristics of the underlying distribution.

For example, for any sample size and *π*_1_ = *π*_2_, *α*_1_ ≠ *α*_2_, and *β*_1_ ≠ *β*_2_, power is greater when the overall proportion of zeros is lower. In these cases, the first bin which contains all the zero observations has equal probability for both samples but the remaining bins profiles are unequal. Thus scenarios with larger bins corresponding to unequal profiles have the greatest power. Simulations where *π*_1_ = *π*_2_ = 0.1 always have greater power than *π*_1_ = *π*_2_ = 0.2 because the bins with unequal profiles have larger relative proportions within the contingency table. Similarly, for any sample size and *π*_1_ = 1/2*π*_2_, α_1_ = α_2_, and *β*_1_ = *β*_2_ (the ratio of zeros is constant as well as non-zero distribution), power is greater when the overall proportion of zeros is greater. In cases where and *π*_1_ ≠ *π*_2_, *α*_1_ ≠ *α*_2_, and *β*_1_ ≠ *β*_2_, the relationship remains but is complicated by differences in the row profiles corresponding to each bin. If we examine situations where the ratio of *π*_1_ and *π*_2_ are equal (*π*_1_ = 1/2*π*_2_) with unequal non-zero profiles, we still observe greater power with greater *π̄* for the parameters in Table 3. Essentially, the difference in the profiles of the bin of zeros is greater than the differences in the non-zero bins.

The results of simulations with zero-inflated Poisson distributions (Table 4) for testing *Q* = (1, 25, 50, 75) show the same characteristics as when gamma distributions are used. The procedure is unchanged as we are dealing with percentiles. However, some distributions with some non-unique values (such as Poisson with small means) may cause some irregularities in the contingency table resulting in irregular jumps in the cumulative distribution. For truly continuous data, such as the simulated gamma samples, each value is unique and the bins within the contingency table will be proportional to the spacing of the percentiles in *Q*. For discrete data, the observations cannot be individually separated because there are several observations with the same value. This does not pose a problem when performing the chi-square test on the contingency table data, but may cause unexpected bin sizes.

## 4. Illustrative Examples

### 4.1. Urinary Triclosan

Urinary triclosan data from the 2011–2012 National Health and Nutrition Examination Surveys (NHANES) were used to illustrate the use of the percentile test with zero inflated distributions. Specifically, we examined the measurements of adult, non-Hispanic white and black participants between the ages of 18 and 79. Triclosan is a broad-spectrum phenolic biocide used in toothpastes, cleaning supplies, and personal-care products. Its use in consumer products has recently been investigated due to potential safety concerns. In experimental animal models, triclosan has been reported to alter hormones [13] [14], although evidence for adverse effects in humans is limited [15]. However, Lankester *et al*. [16] found that urinary triclosan was associated with elevated body mass index.

The lower detection limit (LDL) of urinary triclosan for this laboratory method is 2.3 nanograms per milliliter (ng/ml). Per NHANES procedures, any measurement less than the LDL is replaced with an imputed value of the LDL divided by the square root of two. For urinary triclosan this results in a point distribution at 1.63 mixed with continuous values above the detectable limit. Suppose one was interested in examining differences in the distribution of urinary triclosan between independent populations. The difference in proportion of non-detectable measurements coupled with differences in the detectable measurements would be of interest. For illustrative purposes, consider testing the homogeneity of percentile profiles of independent groups: 1) black females and white females and 2) black males and white males. To test the homogeneity of the percentile profiles, one must follow the steps in Section 2 with the added percentile to account for “zeros”, which in this case is 1.63. Since triclosan is a potentially harmful substance, we are particularly interested in percentiles close to 100. We chose to test for homogeneity of the 1^st^, 50^th^, 60^th^, 70^th^, 80^th^, and 90^th^ percentiles (with the 1^st^ percentile used to test proportion of observations below detection). The combined samples have roughly 25% undetectable measurements so any percentile less than the 25^th^ is adequate for *Q_z_*.

The contingency tables for comparing races with respect to gender-specific triclosan differences are shown in Table 5 and Table 6, respectively. There is a significant difference in the profiles between black and white females (*χ*^2^ = 13.3, df = 6, p = 0.039) as well as black and white males (*χ*^2^ = 13.2, df = 6, p = 0.040). We can conclude that there is no difference between races in the proportion of observations with undetectable amounts of triclosan (bin 1) for both males and females; all observed counts are not significantly different than the expected value under *H*_0_.

If one compares the observed values with the expected (in parentheses), the differences between the groups are not as straightforward as a shift in the distribution. When dealing with large populations with diverse participants and behaviors, such as NHANES, multimodal distributions are expected; other tests may not detect the subtleties that such data often contain. With this method, the analyst can test for differences between groups with greater control and detect differences in specific regions of the distribution.

### 4.2. Serum Cotinine

Serum cotinine data from the 2011–2012 NHANES were used as a second example to illustrate the procedure. Cotinine is the primary metabolite of nicotine and is currently regarded as the best biomarker of tobacco smoke exposure, for both active smoking as well as “passive smoking” [17]. The imputed value for serum cotinine below the LDL for NHANES data is 0.011. Thus we have a point distribution at 0.011 mixed with values greater than 0.011. As with triclosan, we restrict our analysis to adult, non-Hispanic white and black participants between the ages of 18 and 79. The same percentile profile consisting of the 1^st^, 50^th^, 60^th^, 70^th^, 80^th^, and 90^th^ percentiles were tested for homogeneity between: 1) black females and white females and 2) black males and white males.

Results of the percentile test indicate significant differences between percentile profiles for both within gender comparisons. The profiles of black females and white females are significantly different (*χ*^2^ = 75.9, df = 6, p < 0.0001) with the proportion of black females below the detection limit lower than expected and the proportion of white females higher than expected (Table 7). The profiles of black males and white males are also significantly different (*χ*^2^ = 61.7, df = 6, p < 0.0001) with the proportion of black males below the detection limit lower than expected and the proportion of white males higher than expected (Table 8). A separate analysis removing values equal to 0.011 also indicated significant differences when testing *Q* = (50, 60, 70, 80, 90). The non-zero distribution of females (*χ*^2^ = 33.7, df = 5) and males (*χ*^2^ = 15.8, df = 5) were found to be nonhomogeneous between black and white.

**Figure 1:**
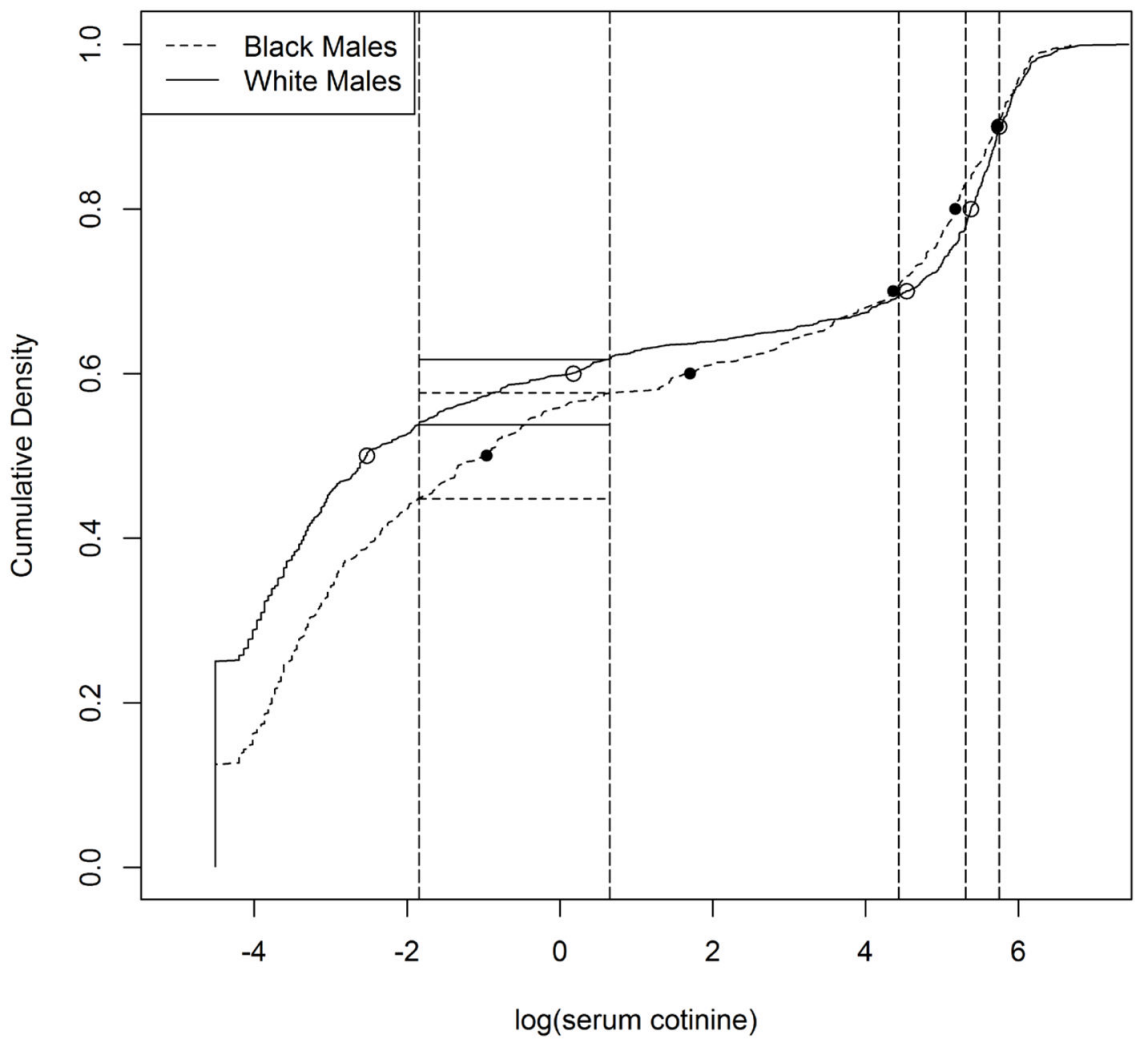
Cumulative distribution functions of log (serum cotinine) for black and white males, including dots for the respective 50^th^, 60^th^, 70^th^, 80^th^, and 90^th^ sample percentiles. Vertical lines indicate location of combined sample percentiles.

The cumulative distribution of log (serum cotinine) for black and white males is plotted in Figure 1. The points on each line are the respective 50^th^, 60^th^, 70^th^, 80^th^, and 90^th^ percentiles for the two groups with short-dashed line representing black males and solid line for white males. The vertical long-dash lines are placed at the value of the combined sample percentile estimates and are the cutoff points for the contingency table. We see that both groups start at the respective proportion of values equal to 0.011, with considerable difference between the two. Although the sample sizes are unequal between the groups, they are scaled equally between 0 and 1 as would any cumulative distribution. By placing horizontal lines (dashed for black males and solid for white males) between two of the cutoff points, we see a graphical representation of the contingency table and the test in general. The number of observations in a bin is the number of observations between two combined sample percentile estimates.

Equivalently, it is the proportion of the cumulative distribution between these two values multiplied by the sample size. The width is the proportion of the sample between the vertical lines. Essentially, we are testing the equality of widths (the change in the cumulative distribution) between a set of combined sample percentile estimates.

## 5. Concluding Remarks

When dealing with zero-inflated data it is useful to compare both the proportion of zero values and the shape of the non-zero values, as measured by a percentile profile. Zero-inflated distributions are frequently encountered in biomedical studies which typically require some hypothesis testing of the equality of the distributions or equality of specific parameters, such as the median. With the proposed procedure, the analyst is able to simultaneously test for differences in the proportion of zeros, along with differences in any number of percentiles selected by the analyst. This flexibility allows the analyst to select the percentiles that best characterize the data and is especially useful for comparing asymmetric or multimodal distributions mixed with one or more point distributions. We find this procedure to be straightforward and easily implemented. It is also appropriate to use when the distributions have unusual shapes.

The proposed procedure has several other advantages when compared to other tests for homogeneity of zero-inflated distributions: 1) the test is nonparametric and can be used for any unimodal or multimodal continuous or discrete distribution; 2) the test allows for multiple point distributions of any value, not necessarily zero exclusively; and 3) it can be used to test for homogeneity of more than two groups simultaneously. As seen in the illustrative examples, the method can distinguish important differences between distributions of populations. For applications with large sample sizes, such as NHANES data, the procedure is particularly useful in testing equality of many percentiles simultaneously.

A limitation is that the test relies on large sample theory and further study is needed to evaluate the severity of this restriction. Simulations show that empirical alpha is adequate by sample size 50 for comparisons of three percentiles in addition to the proportion of zeros; however, the minimum sample size required to achieve the desired alpha is dependent upon the number and choice of percentiles. It is important to remember that there are more powerful tests to test overall equality of distributions (Wilcoxon, KS test) or specific changes in parameters (t-test, ANOVA), However, none of these tests are appropriate for identifying specific segments of distributions that are significantly different. Further research could be done on deriving a closed-form solution for the power of the percentile test for a given percentile profile based on the features of the samples, such as the probability of zeros, the sample size, and the underlying distributions.

## Figures and Tables

**Table 1:** Example of contingency table for testing homogeneity of a three percentile profile.

Sample	Bin 1	Bin 2	Bin 3	Bin 4	Total
1	66	59	48	47	220
2	35	50	49	62	196
3	55	47	59	47	208
Total	156	156	156	156	624

**Table 2:** Example of contingency table for testing homogeneity of a three percentile profile with added bin for zeros.

Sample	Bin 1	Bin 2	Bin 3	Bin 4	Bin 5	Total
1	15	51	59	48	47	220
2	28	7	50	49	62	196
3	42	13	47	59	47	208
Total	85	71	156	156	156	624

**Table 3:** Power simulations for testing *Q* = (1, 50, 75, 90) with zero-inflated gamma distributions.

Sample Size (*n* = *m*)	*π*_1_	*π*_2_	Gamma Distribution Parameters			
			*α*_1_ = 2 *β*_1_ = 2	*α*_1_ = 2.2 *β*_1_ = 2.2	*α*_1_ = 2.3 *β*_1_ = 2.3	*α*_1_ = 2.4 *β*_1_ = 2.4
50	0.1	0.1	**0.0467**	0.1301	0.2536	0.4214
		0.2	0.1605	0.2575	0.3918	0.5512
		0.3	0.4914	0.5865	0.6786	0.7845
100	0.1	0.1	**0.0511**	0.2349	0.4961	0.7627
		0.2	0.3089	0.5310	0.7169	0.8843
		0.3	0.8419	0.9103	0.9582	0.9832
200	0.1	0.1	**0.0500**	0.4684	0.8387	0.9785
		0.2	0.5975	0.8551	0.9672	0.9969
		0.3	0.9938	0.9982	0.9998	1.0000
500	0.1	0.1	**0.0527**	0.8954	0.9987	1.0000
		0.2	0.9619	0.9994	1.0000	1.0000
		0.3	1.0000	1.0000	1.0000	1.0000
50	0.2	0.2	**0.0476**	0.1212	0.2326	0.3845
		0.3	0.1211	0.2033	0.3123	0.4592
		0.4	0.3719	0.4598	0.5620	0.6719
100	0.2	0.2	**0.0493**	0.2140	0.4599	0.7229
		0.3	0.2178	0.4140	0.6173	0.8144
		0.4	0.7015	0.8032	0.8920	0.9539
200	0.2	0.2	**0.0508**	0.4294	0.7991	0.9691
		0.3	0.4218	0.7373	0.9243	0.9893
		0.4	0.9585	0.9878	0.9967	0.9997
500	0.2	0.2	**0.0503**	0.8592	0.9971	1.0000
		0.3	0.8459	0.9932	1.0000	1.0000
		0.4	1.0000	1.0000	1.0000	1.0000

**Table 4:** Power simulations for testing *Q* = (1, 50, 75, 90) with zero-inflated Poisson distributions.

Sample Size (*n* = *m*)	*π*_1_	*π*_2_	Poisson Distribution Parameters			
			*λ*_1_ = 5 *λ*_2_ = 5	*λ*_1_ = 5.5 *λ*_2_ = 5	*λ*_1_ = 6 *λ*_2_ = 5	*λ*_1_ = 6.5 *λ*_2_ = 5
50	0.1	0.1	**0.0465**	0.0876	0.2476	0.5205
		0.2	0.1561	0.2080	0.3850	0.6307
		0.3	0.4850	0.5472	0.6791	0.8342
100	0.1	0.1	**0.0510**	0.1489	0.5009	0.8671
		0.2	0.2944	0.4472	0.7256	0.9458
		0.3	0.8241	0.8839	0.9578	0.9937
200	0.1	0.1	**0.0520**	0.2588	0.8443	0.9960
		0.2	0.5757	0.7803	0.9692	0.9995
		0.3	0.9911	0.9974	0.9996	1.0000
500	0.1	0.1	**0.0476**	0.6354	0.9986	1.0000
		0.2	0.9549	0.9954	1.0000	1.0000
		0.3	1.0000	1.0000	1.0000	1.0000
50	0.2	0.2	**0.0496**	0.0867	0.2345	0.4736
		0.3	0.1187	0.1755	0.3174	0.5459
		0.4	0.3757	0.4284	0.5616	0.7326
100	0.2	0.2	**0.0504**	0.1426	0.4624	0.8281
		0.3	0.2111	0.3362	0.6256	0.8935
		0.4	0.6941	0.7737	0.8940	0.9763
200	0.2	0.2	**0.0502**	0.2526	0.7978	0.9910
		0.3	0.4098	0.6332	0.9326	0.9972
		0.4	0.9596	0.9771	0.9978	1.0000
500	0.2	0.2	**0.0501**	0.6168	0.9978	1.0000
		0.3	0.8422	0.9719	1.0000	1.0000
		0.4	1.0000	1.0000	1.0000	1.0000

**Table 5:** Contingency table for testing homogeneity of *Q* = (1, 50, 60, 70, 80, 90) of urinary triclosan for females.

Females	Bin						
	1 ≤1.6	2 (1.6, 7.2]	3 (7.2, 13.6]	4 (3.6, 23.9]	5 (23.9, 84.3]	6 (84.3, 258]	7 >258
Black Females	58 (63.3)	58 (46.2)	23 (21.4)	17 (21.8)	21 (21.8)	15 (21.8)	26 (21.8)
White Females	90 (84.7)	50 (61.8)	27 (28.6)	34 (29.2)	30 (29.2)	36 (29.2)	25 (29.2)

**Table 6:** Contingency table for testing homogeneity of *Q* = (1, 50, 60, 70, 80, 90) of urinary triclosan for males.

Males	Bin						
	1 ≤1.6	2 (1.6, 5.9]	3 (5.9, ≤9.5]	4 (9.5, 19.9]	5 (19.9, 53.4]	6 (53.4, 173]	7 >173
Black Males	68 (68.5)	52 (53.6)	24 (22.5)	24 (24.3)	33 (23.9)	25 (23.9)	15 (24.3)
White Males	84 (83.5)	67 (65.4)	26 (27.5)	30 (29.7)	20 (29.1)	28 (29.1)	39 (29.7)

**Table 7:** Contingency table for testing homogeneity of *Q* = (1, 50, 60, 70, 80, 90) of serum cotinine for females.

Females	Bin						
	1 ≤0.011	2 (0.011, 0.04]	3 (0.04, 0.09]	4 (0.09, 0.57]	5 (0.57, 68.9]	6 (68.9, 234]	7 >234
Black Females	134 (192)	158 (152)	86 (68)	93 (69)	93 (69)	58 (69)	64 (69)
White Females	291 (233)	179 (185)	65 (83)	59 (83)	59 (83)	94 (83)	88 (83)

**Table 8:** Contingency table for testing homogeneity of *Q* = (1, 50, 60, 70, 80, 90) of serum cotinine for males.

Males	Bin						
	1 ≤0.011	2 (0.011, 0.16]	3 (0.16, 1.93]	4 (1.93, 84.5]	5 (84.5, 202]	6 (202, 313]	7 >313
Black Males	78 (123)	201 (188)	80 (62)	81 (62)	77 (62)	47 (63)	59 (62)
White Males	217 (172)	249 (262)	69 (87)	68 (87)	72 (87)	104 (88)	88 (86)
